# Direct access CT coronary angiography in patients referred with suspected cardiac chest pain: a novel patient pathway

**DOI:** 10.1136/openhrt-2025-003948

**Published:** 2026-04-17

**Authors:** Faheem Ahmad, Jessica Galvin, Wendy Cruickshanks, Laura Miller, Andrew Hunter, David Murdoch, Ross T Campbell, Eugene Connolly, Kenneth Mangion

**Affiliations:** 1 Department of Cardiology, Queen Elizabeth University Hospital, Glasgow, UK; 2 School of Cardiovascular and Metabolic Health, University of Glasgow, Glasgow, UK; 3 Department of Radiology, Golden Jubilee National Hospital, Clydebank, UK

**Keywords:** Computed Tomography Angiography, Coronary Angiography, Coronary Artery Disease, Angina Pectoris, Chest Pain

## Abstract

**Background:**

Rapid access chest pain (RACP) clinics are designed to expedite cardiac assessment, but current pathways cause delays due to sequential consultations and testing. This pilot evaluated a novel direct-to-CT coronary angiography (CTCA) pathway to test the hypothesis that a ‘test-first’ model would reduce the time to diagnosis and clinic utilisation.

**Methods:**

This was a prospective single-centre pilot of consecutive primary care referrals to an RACP service (June 2024–January 2025). Eligible patients with suspected anginal symptoms, no known coronary artery disease (CAD) and no contraindications to CTCA were offered an opt-in to a direct-to-CTCA pathway. CTCA was performed using a prospective single-heartbeat acquisition. The primary outcome was referral-to-diagnosis interval. Secondary outcomes included need for face-to-face consultation, further investigations, incidental findings and theoretical cost savings.

**Results:**

149 patients (mean age 57±9 years, 34% female) underwent CTCA. Median referral-to-diagnosis interval was 29 days (IQR 21–41) vs 88 days (IQR 84–101) in the conventional pathway. CTCA revealed no or mild CAD in 104 (70%) patients; only 47 (32%) required subsequent face-to-face review. Follow-up testing included exercise ECG (17%), echocardiography (8%) and invasive coronary angiography (7%). Incidental findings were uncovered in 30%, with 3% leading to specialty referral. An estimated 102 outpatient visits were avoided, with a cost avoidance estimate of £32 620 per year.

**Conclusions:**

A direct-to-CTCA pathway for patients with suspected cardiac chest pain is feasible, reduces time to diagnosis and aligns with National Institute for Health and Care Excellence guidelines. The pathway enables early CAD exclusion in most patients, reduces unnecessary clinic visits and optimises resource use without compromising diagnostic quality.

**WHAT IS ALREADY KNOWN ON THIS TOPIC**

- National Institute for Health and Care Excellence (NICE) recommends CT coronary angiography (CTCA) as first-line investigation for suspected coronary disease, but current pathways use sequential consultation and testing leading to diagnostic delays.

**WHAT THIS STUDY ADDS**

- A direct-to-CTCA pathway reduced median time to diagnosis from 88 days to 29 days and safely excluded significant disease in 70% of patients, avoiding 102 outpatient visits over 7 months.

**HOW THIS STUDY MIGHT AFFECT RESEARCH, PRACTICE OR POLICY**

- This pilot supports that test-first pathways can implement NICE guidance at scale, thereby optimising cardiology capacity while enabling earlier targeted therapy or disease exclusion.

## Introduction

 Rapid access chest pain (RACP) clinics were established in the UK in 2000 and further mandated in National Health Service (NHS) frameworks for coronary artery disease (CAD).^1^ These mandated the establishment of clinics to allow faster access to specialist cardiology expertise and testing ideally ‘*within two weeks of referral from primary care*’^2^.

Conventional chest pain pathways involve consultation and investigation in a sequential manner, a design that can introduce avoidable delays and inefficiencies in healthcare settings with restricted access to testing. Although prompt access along these routes can lower cardiovascular mortality and emergency department attendances, robust evidence from high-quality randomised controlled trials is still lacking to define the most effective service configuration.^3 4^ In current UK practice, patients referred from primary care are usually directed to a comprehensive ‘one-stop’ clinical assessment. Historically, exercise treadmill testing (ETT) formed the cornerstone of the diagnostic work-up for suspected CAD; however, the National Institute for Health and Care Excellence (NICE) has since relegated ETT due to its modest diagnostic performance, favouring contemporary imaging-based strategies instead.^1 5 6^

Non-invasive investigations, such as CT coronary angiography (CTCA) or stress imaging, are arranged following this consultation, often leading to multiple hospital visits and delayed diagnosis. NICE guidelines have suggested CTCA as the first-line test for patients with new-onset chest pain and no pre-existing coronary disease, regardless of whether symptoms are typical, atypical or non-anginal.^7^ By providing a detailed anatomical assessment, CTCA can exclude CAD in patients with normal coronary arteries.

Recent technical advances—in particular single heartbeat acquisition—have further enhanced diagnostic accuracy of CTCA while improving image clarity.^4^ Unlike earlier multibeat helical protocols that were prone to motion artefact and delivered higher doses, single-beat imaging captures the entire heart in one cycle, usually with radiation exposures below 2 mSv and without loss of sensitivity or negative predictive value.^8 9^

These pathways remain significantly congested; chest pain is estimated to account for up to 1% of all primary care appointments.^10^ In the first year following a primary care consultation for chest pain, the risk of mortality is doubled from baseline.^11^ NHS cardiovascular waiting lists have increased by almost 40% since the coronavirus pandemic (with associated increased morbidity), compounding the need to introduce more efficient referral pathways to restore a functioning service.^12^

In the post-pandemic era, areas of the NHS struggle to meet the 2-week referral-to-clinic target for stable chest pain. To date, there is one retrospective study assessing and confirming that a primary care-led CTCA pathway is feasible and safe. We tested a novel ‘CT-first’ pathway to leverage the high sensitivity and negative predictive value of modern CTCA to rule out coronary disease rapidly, mirroring the ‘test-first, then clinic’ model adopted in oncology pathways to streamline care.^13^

## Methods

**Figure 1:**
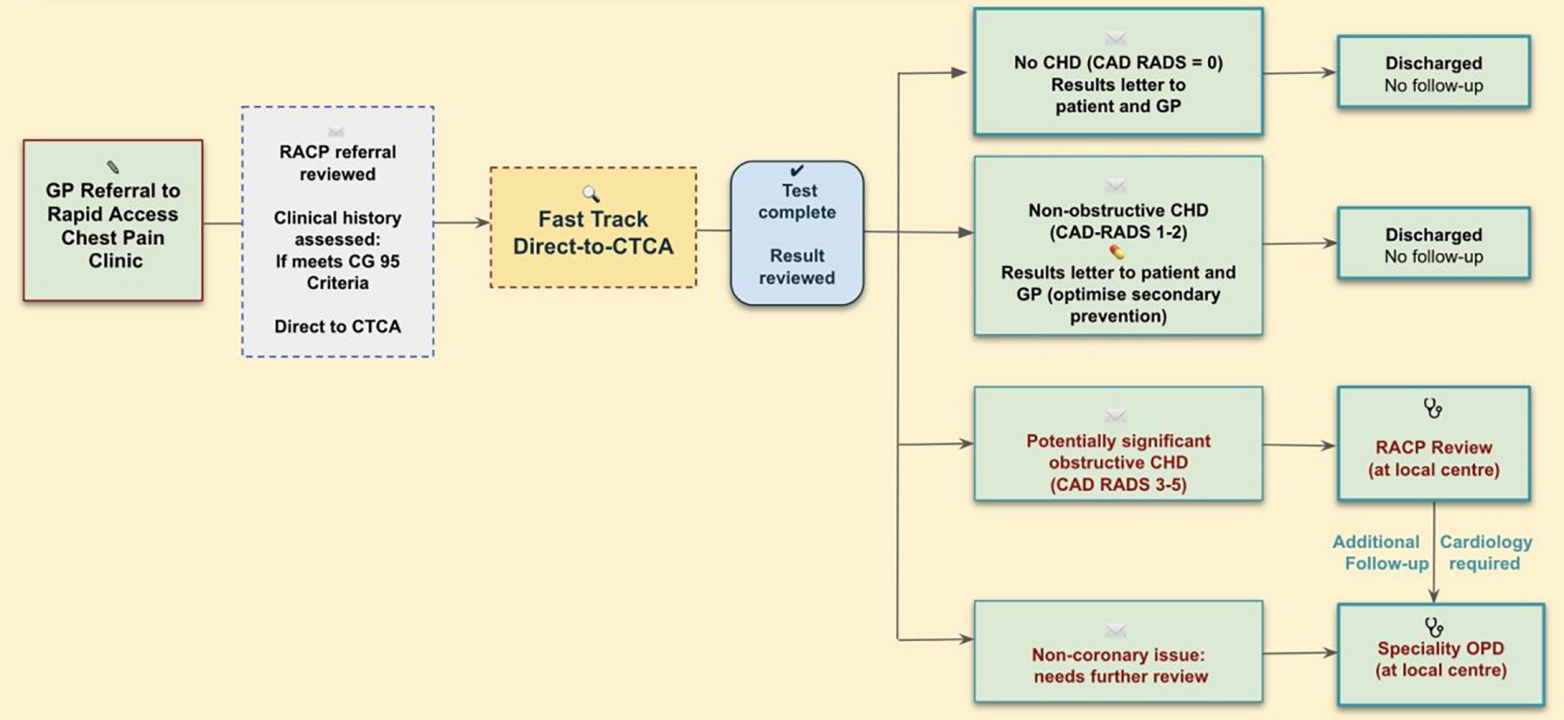
Fast-track CTCA pilot pathway (high-level overview). CAD-RADS, Coronary Artery Disease-Reporting and Data System; CHD, coronary heart disease; CTCA, CT coronary angiography; GP, general practitioner; OPD, outpatient department; RACP, rapid access chest pain.

We conducted a prospective single-centre assessment of consecutive primary care referrals to an urban academic medical centre serving a population of ~650 000 (5 June 2024–22 January 2025) (figure 1). Patient and public representatives reviewed the protocol and application. Cardiologists (EC, FA) screened primary care referrals against NICE guidelines to select patients for a *direct-to-CTCA* approach. An additional weekly CTCA scanning session was used for this pilot study. Standard letters were sent to the general practitioner (GP) at the point of vetting (providing general guidance on beta-blocker introduction). We included patients with typical or atypical anginal chest pain, or non-anginal chest pain with abnormal resting ECG findings. Exclusion criteria included pregnancy, known CAD, contraindications to CTCA (eg, end-stage renal disease, contrast allergy), uncontrolled arrhythmias or a heart rate unsuitable for imaging.

Eligible patients were contacted and offered an opt-in to the pathway and scheduled for CTCA; those who declined remained on the standard RACP pathway. The standard pathway was the comparator group. In the direct-to-CTCA approach, diagnosis was defined as result letter describing whether there was (any) CAD on the CTCA and any actionable outcomes (GP to stop aspirin and statin; clinic review booked; etc). In the comparator arm, diagnosis was defined as the result letter from tests requested at clinic review. The comparator waiting times were taken from just preceding commencement of this test-of-change intervention.

### CT protocol 

Imaging was performed on a 640-detector Canon Aquilion ONE-PRISM scanner. All patients received sublingual glyceryl trinitrate; intravenous metoprolol was titrated to heart rate as per local standard procedure (target ≤60 beats per minute). A prospective single-heartbeat acquisition was used to minimise motion and radiation. Scans were reported by a cardiologist accredited in CTCA (KM), including the Coronary Artery Disease-Reporting and Data System (CAD-RADS).^14^ A radiologist (AH) reviewed non-cardiac anatomy and reported any incidental findings.

### Outcomes

Two cardiologists (FA, KM) reviewed the test results and issued letters. Participants with normal coronary arteries were contacted and informed that they had clear arteries, and any symptoms were likely to be non-cardiac. If minor or mild CAD was detected on CTCA, a standard letter was issued detailing the results and importance of secondary prevention.

Participants with moderate or severe CAD on CTCA were brought to a face-to-face RACP for assessment of symptom burden, optimisation of medication, further testing or onward referral for invasive coronary angiography (ICA).

Incidental cardiac findings such as structural heart disease or arrhythmia had onward testing or general outpatient clinic assessment arranged.

Significant non-cardiac findings were disclosed to patients by a cardiologist, and onward referral to other specialties, or further testing arranged as clinically indicated.

### Data collection and outcomes

Demographics, cardiovascular risk factors, symptoms, CAD-RADS category and subsequent testing were extracted from routine records. The primary endpoint was referral-to-diagnosis interval compared with the contemporary and local RACP pathway. Secondary endpoints included: (1) proportion requiring face-to-face cardiology review after CTCA; (2) additional cardiac/non-cardiac investigations; and (3) outpatient visits avoided and radiation dose.

## Results

**Figure 2:**
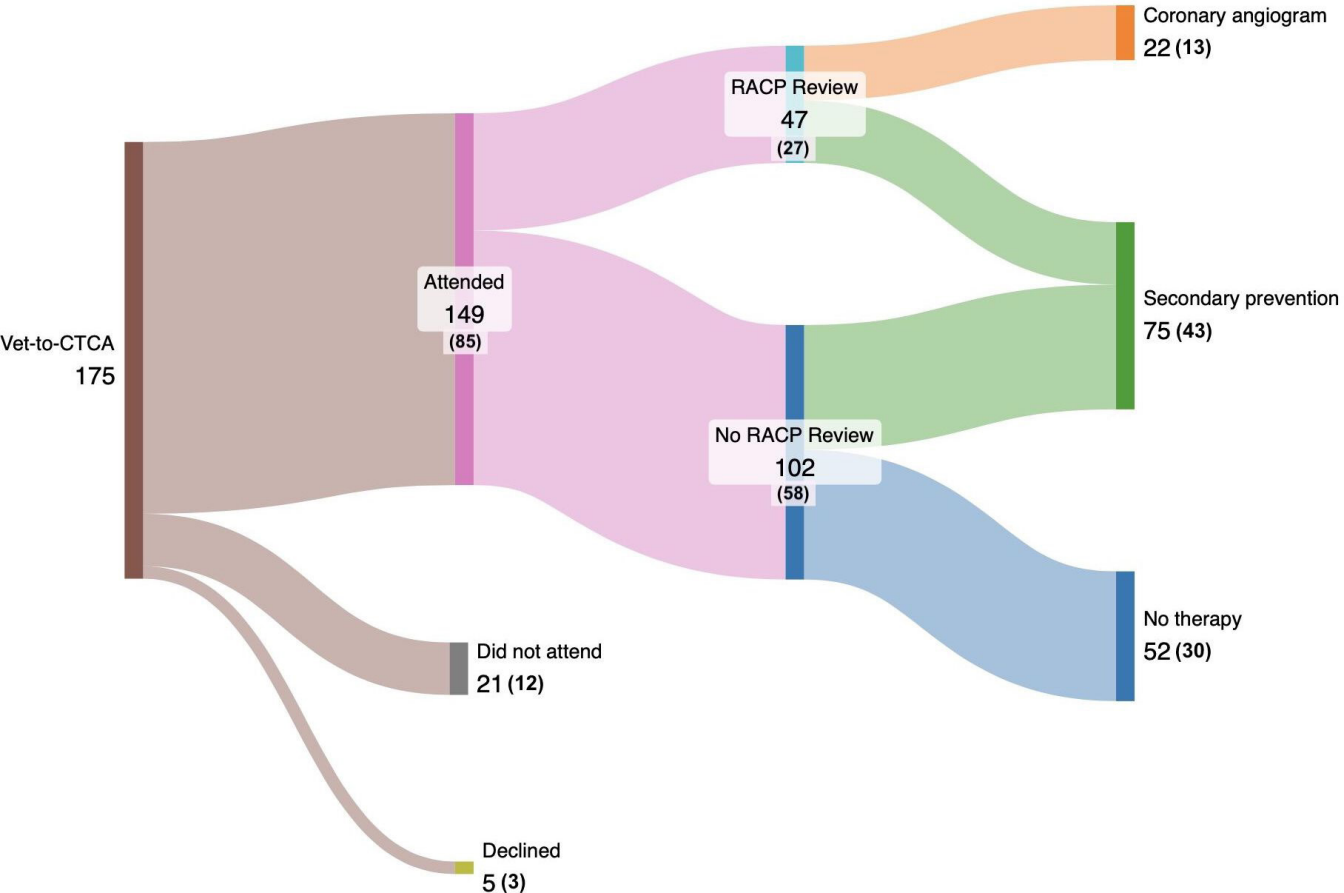
Patient flow during pathway. CTCA, CT coronary angiography; RACP, rapid access chest pain.

149 patients (mean age 57±9 years, 34% female) underwent cardiologist-supervised CTCA (figure 2, table 1). Mean radiation dose-length product was 158±93 mGy/cm with an average intravenous metoprolol dose of 20±20 mg.

**Table 1:** Baseline demographics

	Total (n=149)	Female (n=51)	Male (n=98)
Age (years)	57±9	57±9	55±9
Sex—female (%)	51 (34)	–	–
Smoker (%)	54 (36)	10 (20)	34 (35)
Hypertension (%)	47 (32)	16 (31)	31 (32)
Diabetes (%)	12 (8)	3 (6)	9 (9)
Hypercholesterolaemia (%)	37 (25)	13 (25)	24 (24)
Family history of CAD (%)	54 (36)	24 (47)	30 (31)
Angina type			
Typical angina*	54 (36)	17 (33)	37 (38)
Atypical angina	74 (50)	27 (53)	47 (48)
Non-anginal chest pain plus abnormal resting ECG	21 (14)	7 (14)	14 (14)
CT findings			
Normal coronary arteries (%)	47 (32)	23 (45)	24 (24)
Mild CAD (%)	57 (38)	18 (35)	39 (40)
Moderate to severe CAD (%)	45 (30)	10 (20)	35 (36)

* As stated in NICE CG95.

CAD, coronary artery disease; NICE, National Institute for Health and Care Excellence.

**Figure 3:**
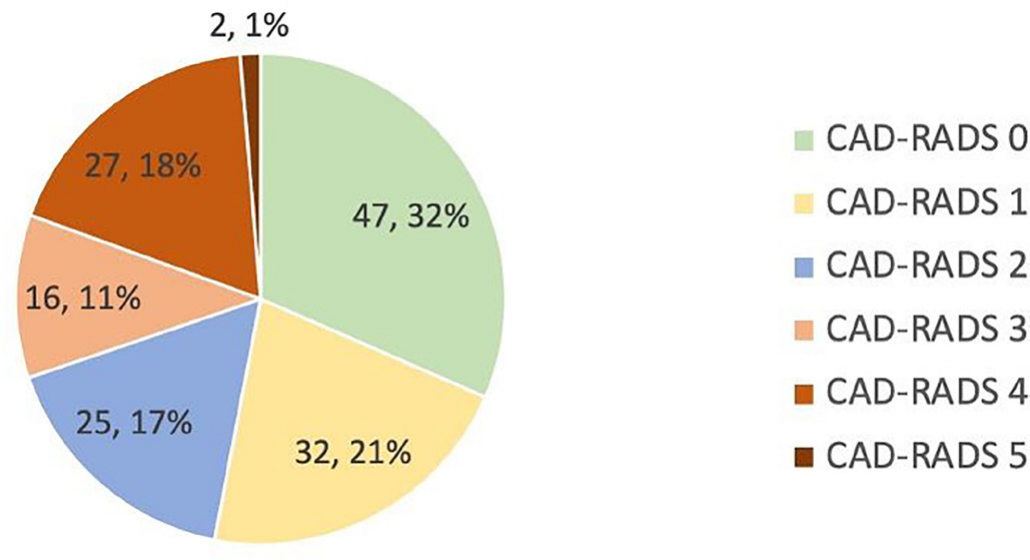
CTCA results by CAD-RADS score (n, %). CAD-RADS, Coronary Artery Disease-Reporting and Data System; CTCA, CT coronary angiography.

The presenting complaints were non-specific chest pain (n=21, 14%), atypical chest pain (n=74, 50%) and typical chest pain (n=54, 36%). 104 (70%) had no or mild CAD by CAD-RADS score (see figure 3). Median referral-to-diagnosis time was 29 days (IQR 21–41) compared with 88 days (IQR 84–101) for patients on the conventional pathway (over the same time period). Further face-to-face RACP consultations were required in 47/149 (32%) of patients.

Follow-on testing comprised exercise ECG in 26 (17 %), transthoracic echocardiography in 12 (8 %) and ICA in 11 (7 %) patients (table 2).

**Table 2:** Follow-on testing

Further management	Total (n=149)	Female (n=51)	Male (n=98)
RACP clinic review (%)	47 (30)	10 (20)	37 (38)
Discharged, no medication (%)	52 (35)	22 (43)	30 (31)
Discharged, medical therapy (%)	97 (65)	29 (57)	68 (69)
Specialty review (respiratory, gastroenterology, %)	4 (3)	0 (0)	4 (4)
Additional testing			
Exercise ECG (%)	26 (17)	5 (10)	21 (21)
Echocardiography (%)	12 (8)	1 (2)	11 (11)
Invasive coronary angiography (%)	11 (7)	3 (6)	8 (8)
CT thorax imaging (%)	7 (5)	1 (2)	6 (6)
Abdominal ultrasound (%)	6 (4)	2 (4)	4 (4)
MRI (%)	1 (1)	0 (0)	1 (1)
Ambulatory ECG (%)	1 (1)	0 (0)	1 (1)

RACP, rapid access chest pain.

Cardiac incidental findings were identified in nine (6%) patients requiring further testing (eight undergoing echocardiography, one cardiac MRI and four referred onwards to a cardiology clinic). Non-cardiac incidental findings were noted in 44 (30%) patients, of which four (3 %) triggered specialty referral for suspected malignancy (online supplemental table 1). Potential non-cardiac causes of symptoms were demonstrated in 18 (12%) patients: hiatus hernia (11, 7%), pulmonary infection (5, 3%), pulmonary embolism (1, 1%) and pulmonary malignancy (1, 1%). All studies yielded adequate image quality; successful diagnostic acquisition was obtained in all patients using prospective single-heartbeat ECG-gated techniques.

During the course of the pilot period, the service avoided 102 outpatient visits. From this, we estimate a potential cost avoidance of £20 196 during the study period, corresponding to an indicative projected annual cost avoidance of £32 620 (outpatient consultation unit cost £230).^15^ These figures are exploratory; a full economic evaluation is required to robustly confirm cost impact.

## Discussion

This prospective evaluation supports that a CTCA-first pathway for stable chest pain referrals can deliver clinical and operational benefits in a resource-constrained healthcare system. By identifying which patients benefited from a CTCA up front before any face-to-face consultation, the median referral-to-diagnosis time was reduced by almost 2 months compared with the conventional RACP pathway. Earlier diagnostic clarification, particularly the confident exclusion of flow-limiting CAD in two-thirds of participants, has several implications: patients gain faster reassurance or targeted therapy; emergency department presentations for unresolved chest pain may fall; and cardiology outpatient capacity is released for higher acuity cases.

Our findings are in keeping with large randomised controlled SCOT-HEART (Scottish COmputed Tomography of the HEART) and PROMISE (Prospective Multicenter Imaging Study for Evaluation of Chest Pain) trials, which showed that an anatomical first-line strategy improves diagnostic accuracy, reduces downstream test requirements and facilitates preventive therapy.^16 17^ In our cohort, only 7% proceeded to ICA, a rate comparable with SCOT-HEART (12 %) and PROMISE (11 %), suggesting that modern CT technology coupled with rigorous vetting can reduce unnecessary invasive testing (with its associated morbidity) without missing significant disease. The near-universal adherence to NICE guidelines achieved here contrasts with national audit data, indicating variable guideline implementation, and highlights how pathway redesign can convert policy into practice.^7^

Rapid exclusion of CAD is clinically important as most individuals referred with chest pain ultimately have a non-coronary aetiology, particularly women and younger patients who are disproportionately misclassified by functional testing.^18 19^ Within this pathway, 32% showed no visible plaque (CAD-RADS 0), allowing immediate discharge and reassurance after a single encounter. A further 38% of the cohort had non-obstructive coronary plaque; these patients were channelled into structured risk factor optimisation, facilitating institution of early antilipid therapy in subclinical atherosclerosis otherwise undetected with classical stress testing, thereby lowering future cardiovascular events.^17^

The high prevalence of zero to mild CAD may indicate a subset of patients may have been at sufficiently low pretest risk to defer imaging. Implementing pretest risk stratification tools could optimise patient selection further. However, the CT-first approach aligns with current guidelines.

At a service level, the pathway obviated the need for 102 (58%) outpatient visits, extrapolating to a projected net yearly saving of £32 620, even before accounting for downstream reductions in functional imaging and coronary angiography. However, given the constrained slot capacity within the pilot pathway, a full-scale implementation would likely result in a greater number of outpatient appointments avoided and additional estimation of cost savings. Further formal research and health economic analyses are required to elucidate these potential benefits more clearly. These gains were achieved without compromising image quality: single heartbeat acquisition delivered diagnostic studies at a mean dose-length product of 158 mGy/cm (≈1.9 mSv). Such performance supports broader NHS ambitions to increase CTCA throughput while adhering to the As Low As Reasonably Achievable radiation principles.

Successful deployment hinged on three operational elements. First, centralised vetting by dedicated cardiologists or personnel filtered out contraindications (eg, renal failure, uncontrolled atrial fibrillation) and initiated set preparatory beta-blocker advice. Second, automated communication with primary care ensured premedication was instituted appropriately (analogous to bowel preparation letters used in colorectal pathways). Third, close cardiology–radiology collaboration allowed real-time optimisation of heart rate and contrast protocols (through delivery of intravenous beta-blocker immediately before imaging), driving down non-diagnostic scans. These components are readily transferable, yet require investment in scanner capacity, trained reporting staff and digital infrastructure—limitations that disproportionately affect rural or resource-constrained regions.^20^

Nearly one-third of scans revealed extracardiac abnormalities; however, most of these were non-significant findings. In 12% of cases, these abnormalities helped inform the possible underlying aetiology of chest pain (such as hiatus hernia, early pneumonia or pulmonary embolism), which would have been missed by stress testing alone. Importantly, only 3% of CTCA cases necessitated specialist referral. The potential for unnecessary additional downstream testing underscores the need for robust triage algorithms—the majority of incidental findings, like pulmonary nodules, typically do not require extensive investigation.^21^ Structured reporting templates that flag clinically actionable lesions and provide follow-up interval (akin to the British Society of Thoracic Imaging nodule guidance) can avoid excess downstream resource utilisation.

Scaling this pathway would require a systems-level approach,^22 23^ particularly in areas where there is limited access to CTCA, such as in regions within Scotland.^22 23^ Community diagnostic centres equipped with cardiac-capable CT, mobile scanners for remote regions and telereporting networks could democratise access. Decision support algorithms within electronic referral platforms could automate guideline checks, flag contraindications and generate prescan prescriptions, further reducing clinical friction.^24^ Patients exhibiting a phenotype of non-obstructive yet diffuse multivessel plaque are likely to require intensified risk factor management; whether this care is delivered in primary or secondary settings should be determined by local commissioning arrangements.

### Limitations

The single-centre design of this pilot, together with strict inclusion and exclusion criteria and the absence of randomisation, introduces referral and selection bias; however, the inclusion of consecutive referrals and comparison with historic RACP performance help mitigate this concern. CT-derived fractional flow reserve was not available in this pilot, which would have further helped stratify intermediate coronary lesions, as it is not routinely available for use in NHS Scotland. Follow-up duration and study size were insufficient to capture long-term clinical outcomes such as myocardial infarction or revascularisation, and economic estimates did not include quality-adjusted life years. Approximately 10% of referrals were unsuitable for CTCA, and detailed data on declined or redirected patients were not collected. This may have introduced further selection bias, as patients who declined CTCA may have had a different symptom burden, perceived risk or preference for face-to-face consultation. We did not account for downstream investigation of incidental findings in our cost-avoidance estimate, which might offset cost savings. Upscaling this pathway should incorporate robust health-economic modelling, patient-reported outcomes and stratified analyses by sex and ethnicity.

## Conclusion

We demonstrate that implementing a direct-to-CTCA approach prior to clinic evaluation for patients referred from primary care without established CAD is both feasible and effective. This approach led to earlier diagnoses, improved adherence to NICE guidelines and reduced hospital encounters by effectively triaging suitable patients directly to imaging.

## Supplementary material

10.1136/openhrt-2025-003948 online supplemental table 1

10.1136/openhrt-2025-003948 online supplemental file 1

## Data Availability

Data are available upon reasonable request.
