# Supplementary material for: Direct access CT coronary angiography in patients referred with suspected cardiac chest pain: a novel patient pathway
Source: Open Heart. 2026 Apr 17;13(1):e003948. doi: 10.1136/openhrt-2025-003948 (PMC13110677; doi:10.1136/openhrt-2025-003948)
Supplement: online supplemental table 1 [file openhrt-13-1-s001.docx]

## Supplemental Table: Incidental findings

| Relevant incidental findings |  |
| --- | --- |
| **Cardiac findings** |  |
| Atrial septal defect (%) | 4 (3) |
| Left ventricular hypertrophy (%) | 2 (1) |
| Aberrant coronary artery (%) | 1 (1) |
| Aortic root dilatation (%) | 1 (1) |
| Aortic valve disease (%) | 1 (1) |
| **Non-cardiac findings** |  |
| Hiatus hernia (%) | 11 (7) |
| Hepatic lesions (fatty liver, cysts, nodules) (%) | 11 (7) |
| Pulmonary nodule (%) | 7 (5) |
| Pulmonary infection (%) | 5 (3) |
| Pulmonary fibrosis (%) | 2 (1) |
| Fatty liver (%) | 2 (2) |
| Generalised adenopathy (%) | 1 (1) |
| Pulmonary embolism (%) | 1 (1) |
| Lung Malignancy (%) | 1 (1) |
