# Supplementary figures and images for: Direct access CT coronary angiography in patients referred with suspected cardiac chest pain: a novel patient pathway
Source: Open Heart. 2026 Apr 17;13(1):e003948. doi: 10.1136/openhrt-2025-003948 (PMC13110677; doi:10.1136/openhrt-2025-003948)

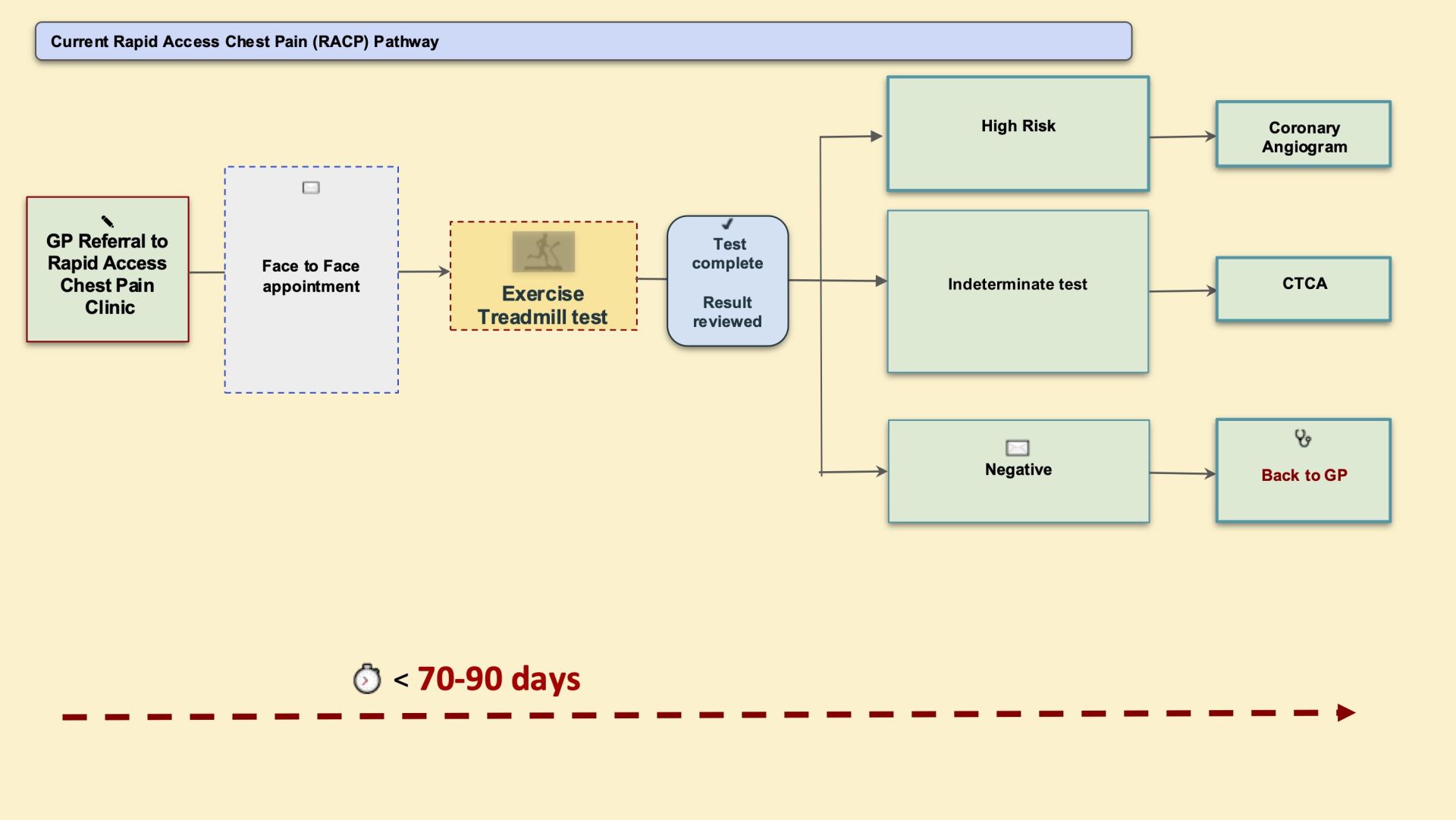

Supplement: online supplemental file 1 [file openhrt-13-1-s002.jpg]
